# Genetic Characterization of Providence Virus Isolated from Bat Guano in Hungary

**DOI:** 10.1128/genomeA.00403-16

**Published:** 2016-05-19

**Authors:** Gábor Kemenesi, Fanni Földes, Brigitta Zana, Kornélia Kurucz, Péter Estók, Sándor Boldogh, Tamás Görföl, Krisztián Bányai, Miklós Oldal, Ferenc Jakab

**Affiliations:** a Virological Research Group, János Szentágothai Research Centre, University of Pécs, Pécs, Hungary; b Institute of Biology, Faculty of Sciences, University of Pécs, Pécs, Hungary; c Department of Zoology, Eszterházy Károly College, Eger, Hungary; d Aggtelek National Park Directorate, Jósvafő, Hungary; e Institute for Veterinary Medical Research, Centre for Agricultural Research, Hungarian Academy of Sciences, Budapest, Hungary; f Department of Zoology, Hungarian Natural History Museum, Budapest, Hungary

## Abstract

We report the complete genome sequence and genetic characterization of a novel strain of Providence virus, detected in *Barbastella barbastellus* bat guano, collected in Hungary in 2014. Our data may facilitate the understanding of the evolutionary processes of this unique viral family of *Carmotetraviridae.*

## GENOME ANNOUNCEMENT

Tetraviruses are classified into three families: *Alphatetraviridae*, *Permutotetraviridae*, and *Carmotetraviridae*, according to the sequence of their viral replicase, which may be alpha-virus-like, picornavirus-like, or carmovirus-like, respectively. Providence virus (PrV) belongs to the genus *Alphacarmotetravirus* and represents the only member within the family *Carmotetraviridae* (1). PrV was originally isolated from a persistently infected *Helicoverpa zeae* MG8 midgut tissue culture cell line and is the only tetravirus to replicate in tissue culture (2). The virus has a positive-sense, single-stranded RNA (ssRNA) genome of 6.4 kb encoding three open reading frames (ORFs), comprising the putative viral replicase (p104) followed by the capsid protein precursor ORF, which is translated from a subgenomic RNA of 2.4 kb. Among tetraviruses, PrV possesses a third ORF at the 5′ end encoding a nonstructural protein of unknown function, overlapping with the replicase gene (3).

In this study, we used viral metagenomics to investigate Western barbastelle bat (*Barbastella barbastellus*) guano samples, collected in multiple localities across Hungary. This study was approved by the National Inspectorate for Environment, Nature and Water (4/2138-7/2011); no animals were invasively sampled or harmed during collection. The strain 14EPF155 was detected in a guano sample derived from a female *B. barbastellus* collected in Nagyvisnyó, Hungary, in September 2014. The genome sequence of PrV (14EPF155) was determined with next-generation sequencing (Ion Torrent PGM) and sequence data were further confirmed via Sanger sequencing. Phylogenetic analysis was performed using the MEGA6 software package (4).

The genomic organization of the Hungarian PrV strain corresponded to the strain vFLM1 isolated in the United States (3). The complete genome of 14EPF155 was 6,158 bp long. All major ORFs were detected (viral replicase p40/p140 with a 413/970-amino acid [aa] length; p81 capsid protein precursor with 754 aa; and p130 nonstructural protein of unknown function with 1,221 aa) along with the read-through stop codon at the termini of p40 replicase. Nucleotide sequence identity compared to the vFLM1 strain was 85% regarding the entire genome, while aa identity was 76% for p130; 91% for p104; 86% for p40; and 87% for the p81 capsid precursor gene. The phylogenetic position of the 14EPF155 PrV strain was monophyletic with the vFLM1 strain, and the Hungarian variant unambiguously clustered within the *Carmotetraviridae* family.

Dietary viruses from bat guano samples were previously described as constituting the majority of viral sequence reads (5, 6), although the original host of these viruses is unpredictable without further examination. A close relative of its original host, namely, the Old World cotton bollworm *Helicoverpa armigera*, which is a significant pest of agriculture in Asia, Europe, Africa, and Australasia (7), may serve as a host. Further studies will be required to decipher the true host of the Hungarian PrV strain.

### Nucleotide sequence accession number.

The GenBank accession number for Providence virus is KU885997.
